# Factors associated with improved survival among older colorectal cancer patients in the US: a population-based analysis

**DOI:** 10.1186/1471-2407-9-227

**Published:** 2009-07-13

**Authors:** Kathleen Lang, Jonathan R Korn, David W Lee, Lisa M Lines, Craig C Earle, Joseph Menzin

**Affiliations:** 1 Boston Health Economics, Inc, Waltham, USA; 2 Health Economics and Outcomes Research, GE Healthcare, Waukesha, USA; 3 Institute for Clinical and Evaluative Sciences, Sunnybrook Health Sciences Center, Toronto, Canada; 4 Harvard Medical School, Boston, USA

## Abstract

**Background:**

The purpose of this study was to estimate the relative impact of changes in demographics, stage at detection, treatment mix, and medical technology on 5-year survival among older colorectal cancer (CRC) patients.

**Methods:**

We selected older patients diagnosed with CRC between 1992 and 2000 from the SEER-Medicare database and followed them through 2005. Trends in demographic characteristics, stage at detection and initial treatment mix were evaluated descriptively. Separate multivariate logistic regression models for colon (CC) and rectal cancer (RC) patients were estimated to isolate the independent effects of these factors along with technological change (proxied by cohort year) on 5-year survival.

**Results:**

Our sample included 37,808 CC and 13,619 RC patients (combined mean ± SD age: 77.2 ± 7.0 years; 55% female; 87% white). In recent years, more CC patients were diagnosed at Stage I and fewer at Stages II and IV, and more RC patients were diagnosed at Stage I and fewer at Stages II and III. CC and RC patients diagnosed in later years were slightly older with somewhat better Charlson scores and were more likely to be female, from the Northeast, and from areas with higher average education levels. Surgery alone was more common in later years for CC patients while combined surgery, chemotherapy, and radiotherapy was more common for RC patients. Between 1992 and 2000, 5-year observed survival improved from 43.0% to 46.3% for CC patients and from 39.4% to 42.2% for RC patients. Multivariate logistic regressions indicate that patients diagnosed in 2000 had significantly greater odds of 5-year survival than those diagnosed in 1992 (OR: 1.35 for CC, 1.38 for RC). Our decomposition suggests that early detection had little impact on survival; rather, technological improvements (e.g., new medical technologies or more effective use of existing technologies) and changing demographics were responsible for the largest share of the change in 5-year survival in CC and RC between 1992 and 2000.

**Conclusion:**

Technological advances and changes in patient demographics had the largest impact on improved colorectal cancer survival during the study period.

## Background

Colorectal cancer (CRC) is the third-most common cancer type in the United States and the third-leading cause of cancer deaths among both men and women[1]. Almost 70% of CRC patients are diagnosed at age 65 years or older. Currently, 5-year relative survival among US CRC patients of all ages is estimated at around 65% and has improved from 59% among patients diagnosed 20 years ago[1]. The prognosis among older CRC patients is substantially worse, with 5-year survival estimated at 47%[2]. Early diagnosis is critical to the chances of survival in CRC, with 5-year survival rates for patients of all ages ranging from 95% for those diagnosed at Stage I to 7% for those diagnosed at Stage IV[3].

Advances in screening technologies (such as flexible sigmoidoscopy, FOBT, and colonoscopy), and an increase in the proportion of the population undergoing regular screening over the past decade may result in lower CRC incidence and a shift toward detection at earlier stages[4]. Medicare's reimbursement policy was changed in 1998 to provide coverage for screening colonoscopies for older patients at increased colon cancer risk, and the policy was expanded in 2001 to cover screening colonoscopies for all Medicare enrollees[5]. Gross and colleagues evaluated shifts in the stage distribution during the years surrounding these policy changes (1992–2002), finding that the percentage of older patients diagnosed at an early stage improved significantly[6]. Technologies such as computed tomography also appear to have led to an improvement in staging accuracy[7].

Most previous studies of survival in colorectal cancer have used older and/or geographically limited data, have focused only on certain stages, subsites, or subgroups in the CRC population, have not focused on older patients, or have used non-US data[8,9]. Additionally, there have been few attempts to disentangle the complicated factors contributing to improvements in survival. Aside from earlier diagnosis, improvements in survival may also reflect a myriad of other factors, such as changes in patient demographics or technological advances in the form of new treatments or "know-how" (ie, more effective use of existing treatments). For example, from the late 1950s until 1996, fluorouracil (5-FU) was the only drug approved for the treatment of colorectal cancer; however, since that time, new targeted chemotherapies and improved surgical techniques likely had positive influences on survival rates[10]. Furthermore, advances in diagnostic techniques that allow for better detection can lead to stage migration and a misinterpretation of improved survival (sometimes referred to as the Will Rogers effect)[11,12].

The objectives of this study were to investigate trends in 5-year survival among older CRC patients and to disentangle the relative influences of four key factors expected to affect changes in survival over time: demographics, early detection, treatment mix, and technological improvements.

## Methods

### Data Source

This study used the linked Surveillance, Epidemiology, and End Results (SEER)-Medicare database, a collaborative effort of the National Cancer Institute, the SEER cancer registries, and the Centers for Medicare and Medicaid Services (CMS). SEER is a surveillance system consisting of population-based tumor registries designed to track incidence and survival in the US. The registries routinely collect information from multiple reporting sources (such as hospitals, outpatient clinics, laboratories, private medical practitioners, nursing and convalescent homes, hospices, autopsy reports, and death certificates) about newly diagnosed cancer patients in geographically defined areas that represent approximately 25% of the US population[13].

Complete details of the linkage of SEER and Medicare data have been previously described elsewhere[14]. Briefly, the linked SEER-Medicare database used in our study includes a SEER file of patients diagnosed with cancer within the geographic areas covered by SEER registries between 1991 and 2000, as well as Medicare claims files covering the period from 1991 through 2005. The SEER files include demographics (eg, age, sex, race, date of death), diagnosis information for up to 10 different incident cancer cases for each person (eg, date of cancer diagnosis, cancer subsite, cancer stage at diagnosis), and indicators for chemotherapy and radiation treatment in the first 4 months after diagnosis. The Medicare administrative claims files include monthly Medicare enrollment data (eg, HMO enrollment, Part A and/or Part B eligibility), individual claims for inpatient and skilled nursing facility (SNF) hospitalizations, outpatient hospital visits and miscellaneous ambulatory services, Part B physician services, home health agency services, and hospice services, including dates of service, ICD-9-CM diagnosis and procedure codes, DRG codes, HCPCS codes, primary insurer payments, patient deductibles and copayments, and Medicare payments for claims. Combining the SEER registry data with the Medicare claims data offers the opportunity to link service utilization over time to stage at diagnosis, as well as the time from diagnosis to death.

### Patient Selection and Follow-up

All patients aged 66 years and older with a new diagnosis of malignant adenocarcinoma of the colon or rectum (ie, presence of a SEER cancer site recode value between 15 and 27 and one of the following ICD-O-3 histology codes: 8140, 8210-11, 8220-21, 8260-63, 8470, 8480-81, or 8490) reported to a SEER registry between January 1, 1992 and December 31, 2000 were identified for possible inclusion in the CRC cohort. Only patients known to be diagnosed at Stages I-IV were included. To ensure that complete data were available for evaluating comorbidities and outcomes, patients were excluded if at any time in the 12-month period before the index date, or anytime after the index date, they were enrolled in a Medicare HMO, not eligible for both Medicare Part A and B benefits, or eligible for benefits under the end stage renal disease program. We also excluded patients who had claims in the 12-month pre-index period consistent with a history of any other cancer, or were diagnosed with CRC at the time of death or autopsy. Finally, since this analysis was conducted as part of a study that evaluated costs among older CRC patients and matched comparison patients[15], we also excluded patients who could not be matched to an appropriate comparator (less than 2% of the sample). The index date for each patient was defined as the date of his or her CRC diagnosis. Patients were followed for 5 years after the index date or until death, whichever came first.

### Statistical Analyses

Study patients were described in terms of baseline demographic and clinical characteristics including age, gender, race, location of residence, cancer stage at diagnosis (Stage I-IV), treatment type (surgery, chemotherapy, radiation, and combinations thereof), and Deyo-Charlson comorbidity score[16] (calculated based on claims during the 12-month pre-index period). Treatments were identified using SEER treatment variables as well as claims data in the 3-month post-index period using ICD-9-CM diagnosis and procedure codes, HCPCS/CPT codes, and revenue center codes (Additional file 1: Table 1). Descriptive analyses of trends in baseline and clinical characteristics and 5-year observed survival (evaluated in terms of the percentage of patients alive 5 years after CRC diagnosis) were conducted.

Separate multivariate logistic regression models of 5-year survival were estimated for CC and RC, controlling for age, sex, race/ethnicity, region, urban/rural residence, education (≤25% of residents in ZIP code with <12 years of education vs. >25%), income (median household income of ZIP code ≤$50,000 vs. >$50,000), stage at diagnosis (specified 2 ways: as individual dummy variables for Stages II-IV and as one dummy variable for Stages II-IV combined, both with Stages I as reference), treatment type (surgery, chemotherapy, radiation, and combinations thereof), and year of diagnosis. Year of diagnosis served as a proxy for changes in medical technology in the regression model.

Results from the logistic regressions were used to model the individual impact of 4 key factors (demographics, early diagnosis, treatment mix, and technology) on observed changes in 5-year survival in CC and RC between 1992 and 2000. We first used estimated coefficients from the logistic regressions to predict 5-year survival in 1992 using average 1992 values for all variables in the regressions. We then generated 4 hypothetical predicted values of 5-year survival in 2000 by changing each of the factors of interest in the model one at a time to their average 2000 value, keeping all other variables at their average 1992 values. The individual impact of each factor was obtained by dividing the relevant hypothetical predicted survival for 2000 by the prediction for 1992. Summing the individual effects yielded an estimate of the overall change in 5-year survival from 1992 to 2000. For the decomposition analysis, we used results from the logistic regression in which stage at diagnosis was specified as early vs. late (ie, Stage I vs. Stages II-IV combined) because the low survival rate among Stage IV patients led to underprediction using the models with individual stage dummy variables.

All analyses were conducted separately for colon and rectal cancer patients. All data analyses were conducted using the Statistical Analysis System (SAS) software package, version 9.1 (Cary, North Carolina, USA).

## Results

### Patient Characteristics

There were 124,280 patients in the dataset, of which 51,427 met all study inclusion criteria (37,808 CC patients and 13,619 RC patients). Demographic and clinical characteristics for study patients are presented in Additional file 2: Table 2. The mean ± SD age was 77.2 ± 7.0 years and approximately 87% were white, with about 57% of CC patients and 50% of RC patients being female.

**Figure 1:**
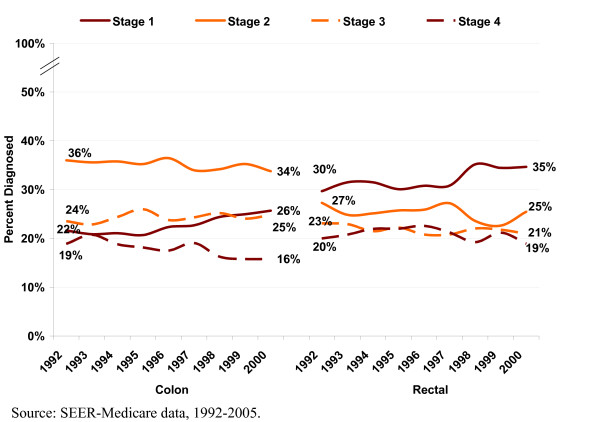
**Stage distribution by year of diagnosis and cancer subsite**.

Approximately 25% of the overall CRC population was diagnosed at Stage I, 32% at Stage II, 24% at Stage III, and 19% at Stage IV (Additional file 2: Table 2). RC patients were more likely than were CC patients to be diagnosed at Stages I or IV. In the overall CRC population, the mean ± SD Charlson comorbidity score (not including cancer) was approximately 1.9 ± 1.9. Common comorbidities included chronic pulmonary/respiratory disease, congestive heart failure, and diabetes without complications. Baseline and demographic characteristics did not differ substantially by year of diagnosis, although individual years had some small differences in the Charlson score (slightly declined over the study period), and the distributions of age, region, education levels, and treatment mix (data not shown). CC and RC patients diagnosed in 2000 were about a year older on average, and were slightly more likely to be female, from a zip code with a higher average education level, and from the Northeast than were those diagnosed in 1992. CC patients diagnosed in 2000 were more likely to receive surgery only than those diagnosed in 1992, and RC patients were more likely to receive surgery, chemotherapy, and radiotherapy. Figure 1 illustrates trends in the distribution of stage at diagnosis by year of diagnosis and cancer subsite. Comparing diagnosis years 1992 and 2000, the data show a trend toward more CC patients diagnosed in Stage I and fewer in Stages II and IV. For RC patients, we observed an increase over time in the number of patients diagnosed at Stage I instead of Stages II and III.

### Trends in 5-year Survival

**Figure 2:**
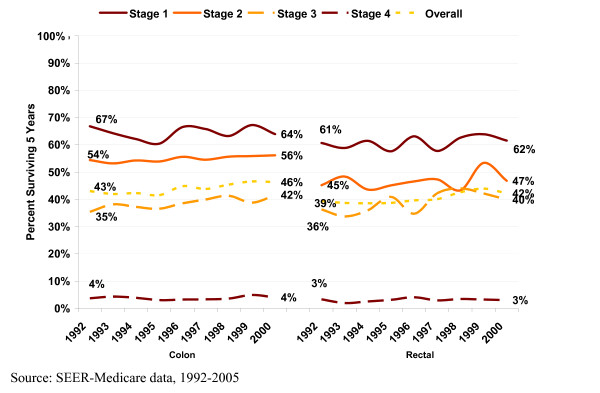
**Five-year survival, by cancer subsite and year of diagnosis**.

Comparing patients diagnosed in 1992 and 2000, overall 5-year survival improved by 7.7% (from 43.0% to 46.3%) for CC patients and by 7.1% (from 39.4% to 42.2%) for RC patients (Figure 2). The largest improvement for both CC and RC patients was in Stage III (35.4% to 41.5% for CC patients, 36.3% to 39.9% for RC patients), while Stage I CC patients and Stage IV RC patients exhibited slight declines in 5-year survival.

### Predictors of 5-year Survival

Multivariate logistic regression results predicting 5-year survival for CC and RC patients, along with corresponding 95% confidence intervals and *P *values, are shown in Additional file 3 Table 3. Among both CC and RC patients, younger patients (66–74 and 75–84 yrs vs. 85+ yrs), women, and those in more educated ZIP codes had significantly better odds of 5-year survival. Patients living in metropolitan counties, those with lower incomes, and those with higher Charlson scores had significantly worse odds of surviving 5 years. Receiving any type of treatment had a large and significant impact on 5-year survival, with patients who received both surgery and chemotherapy having the highest odds of survival in both cohorts. Patients diagnosed in later stages had significantly and progressively worse odds of 5-year survival than those diagnosed at Stage I. Being diagnosed in a later year – our proxy for changes in technology – was also associated with significantly better odds of 5-year survival for both CC and RC patients, with patients diagnosed in 2000 between 1.3 and 1.4 times more likely than patients diagnosed in 1992 to survive 5 years, holding all other variables constant.

### Decomposition of Changes in 5-year Survival

**Figure 3:**
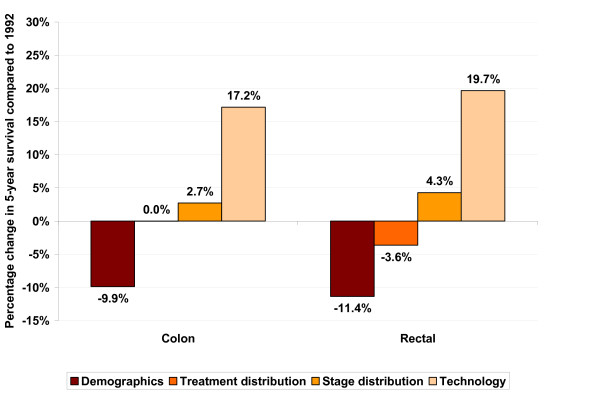
**Estimated changes in 5-year survival from 1992 to 2000 resulting from changes in demographics, treatment, stage distribution and technology**.

Figure 3 presents estimates of the individual impact of changes in demographics, treatment mix, stage at diagnosis, and technology (year of diagnosis) on 5-year survival from 1992 to 2000. Among CC patients, changing only demographics between 1992 and 2000 (and leaving all other variables at their 1992 values) resulted in an estimated 9.9% reduction in predicted 5-year survival. In other words, if patients diagnosed with CRC in 2000 had the same demographic characteristics as their counterparts in 1992, their 5-year survival would have been lower by 9.9%. Changing only the treatment mix yielded no change in survival, while changing only the stage distribution yielded a 2.7% increase. Finally, changing only the year dummy, which is intended as a marker for improvements in technology, to 2000 resulted in a 17.2% increase, by far the largest impact among all factors studied.

Among RC patients, changing only demographics between 1992 and 2000 resulted in an 11.4% reduction in predicted 5-year survival. Changing only the treatment mix yielded a 3.6% reduction, while changing only the stage distribution resulted in a 4.3% increase. Finally, changing only the marker for technology to 2000 led to the highest predicted probability of 5-year survival, a 19.7% increase.

Summing the individual positive and negative impacts of the 4 factors of interest yielded estimated net increases in predicted 5-year survival during the study period of 10.0% in CC and 9.0% in RC. These estimated net increases are somewhat higher than the observed increases in 5-year survival in CC and RC during the study period (ie, 7.7% and 7.1%, respectively), likely resulting from imprecision in the prediction model and additional unmeasured influences that had a small net negative impact on 5-year survival during the study period.

## Discussion

This study evaluated factors affecting the change in 5-year survival among Medicare beneficiaries diagnosed with CC and RC between 1992 and 2000. We found that patients diagnosed in 2000 were about a year older on average, with slightly lower Charlson scores, and were more likely to be female than those diagnosed in 1992. CC and RC patients diagnosed in 2000 were more likely to receive surgery only and surgery, chemotherapy and radiotherapy, respectively, than were patients diagnosed in 1992. We found that there have been considerable changes in stage distribution between 1992 and 2000, and that unadjusted 5-year survival improved by 7.7% (from 43.0% to 46.3%) for CC patients and by 7.1% (from 39.4% to 42.2%) for RC patients. As a comparison, unadjusted 5-year survival for patients in our larger study, who were matched to our CRC patients on age and gender, was 70.9% (unpublished authors' analyses).

Controlling for year of diagnosis and treatment mix, being diagnosed at later stages was shown to have a substantial negative impact on 5-year survival, as we would expect. Similarly, controlling for stage at diagnosis, patients diagnosed in later years (1996–2000) had significantly better survival odds than did patients diagnosed in 1992. In decomposing the effects of demographics, treatment mix, stage at diagnosis and technological change, we found that changes in demographics between 1992 and 2000 had a large negative effect on 5-year survival, while year of diagnosis, which we hypothesized as a marker for technological improvements, had the largest positive effect on 5-year survival. These results suggest that, despite the amount of effort that has gone into screening and colonoscopies, changes in stage distribution have contributed only a small amount to improvements in 5-year survival. This may be due, in part, to a possible bias caused by improved staging. Whereas screening and colonoscopies may have led to finding cancers earlier, improvements in staging accuracy may have contributed to a stage migration effect[11,12].

Our study contributes to the existing literature on survival in colorectal cancer by focusing explicitly on older patients (the primary age group affected by this cancer), and by evaluating the latest available data, thus reflecting some of the recent changes in treatment patterns and detection for CRC. In addition, to our knowledge, ours is the first study that has attempted to disentangle the effects of earlier detection, demographics, treatment mix, and improvements in technology on 5-year survival in CRC patients using logistic regression techniques.

### Comparisons with Other Literature

Despite some methodological differences, our findings are consistent with previous research on changes in stage distribution and survival in CRC. Recently, Gross et al used 11 years of SEER-Medicare data (1992–2002) to evaluate changes in the distribution of stage at diagnosis among colon cancer patients[6]. They compared 3 time periods: before the first change in Medicare's reimbursement policy (1992–1997), between the first and second changes (1998–June 2001), and after the second change (July 2001–2002). They showed that the proportion of CC patients diagnosed at an early stage increased from 22.5% in period 1 to 25.5% in period 2 and 26.3% in period 3 (all significant increases), and our results were nearly identical.

Three previous studies have analyzed increases in US colorectal cancer survival rates over time, all of which examined a wider range of ages than our study. Jemal et al[17] compared 5-year relative survival rates from the mid-1970s to 1995–2000 and found that colorectal cancer had the second-highest improvement in survival rates of all cancer types in that time period. They found that 5-year survival increased among men from 50% to 64% and among women from 52% to 63%. Brenner et al[8] conducted a period analysis of SEER data on 5- and 10-year relative survival and found that both colon and rectal cancer patients had improved survival from 1998 to 2003. They found that for colon cancer patients, 5-year relative survival improved from 63% to 66% and 10-year survival improved from 57% to 62%, and for rectal cancer patients, 5-year relative survival improved from 64% to 67% and 10-year survival improved from 56% to 60%. Finally, Clegg and colleagues[18] generated Kaplan-Meier estimates from SEER data and determined that relative survival had significantly improved from 1988 to 1997. Using similar data (1986–1997 SEER data), another study found that there were no significant improvements in 5-year relative survival[19]. However, this study split the years of interest into 4 groups, concluding that there was no significant improvement in survival from 1986–1988 to 1995–1997.

Our results differ from previous studies for several reasons: 1) our population was limited to older patients; 2) we excluded patients who were enrolled in an HMO, who may have been healthier or had other characteristics that differed from the patients in our sample; and 3) we measured observed, rather than relative, survival. Observed survival takes all causes of death into account, whereas relative survival – the ratio of observed survival to expected survival in a comparable group of cancer-free individuals – is substantially higher than observed survival in older CRC patients[20]. Using cause-specific survival to estimate relative survival requires that the information on cause of death be reliably coded. Even when cause of death is available to a cancer registry via the patient's death certificate, the information is often difficult to interpret. For example, Welch et al[21] studied deaths among cancer patients that occurred within 1 month of diagnosis and found that 41% of deaths were not attributed to the coded cancer.

Few previous studies have attempted to isolate the impact of individual influences on survival in CRC, with most analyzing survival using joinpoint analysis and annual percentage changes instead[3,8]. However, in a recent analysis, Sun and colleagues evaluated the impact of treatment and stage distribution on survival for multiple cancers using SEER data[22]. They found that advances in treatment accounted for the majority of observed changes in survival (78% for all cancers examined) between 1980 and 2000. Since these researchers did not distinguish between treatment mix and technological advances in treatment and did not account for the influence of demographics, it is not possible to compare our results directly. However, their findings are consistent with ours in suggesting that advances in treatment (ie, technology) had the greatest impact on changes in survival over time.

### Limitations

This study is subject to certain limitations that are common to all studies that rely on retrospective claims data, such as potential coding errors and incomplete data[23]. Our use of the SEER-Medicare database, which includes complete claims only for Medicare-eligible patients aged 65 years and older, introduces additional limitations[24,25]. While older patients comprise the vast majority of patients with CRC, this sample is not representative of all US patients, particularly those with other forms of health insurance (eg, managed care, private pay). Despite its limitations, SEER-Medicare data have been used in numerous published studies of colon cancer, as well as cancers of the breast, prostate, and lung, among others[26]. In addition, this study observed improvements in survival among CRC patients that may not be independent of improved survival for the general population. This study relied on year dummy variables to measure changes over time, which required the assumption that all determinants of survival were included and accurately measured in our specification.

Until recently, a combination of fluorouracil (5-FU) and leucovorin was the standard of care for CRC; numerous other drugs have been approved for use in CRC patients since 2004, including oxaliplatin, bevacizumab, cetuximab, and panitumumab[27]. Because our study period ended in 2005, the influence of these new treatments on survival could not be measured. Future researchers will have the ability to use data collected since 2004, which will likely show greater changes in survival attributable to changes in treatment mix. Future researchers will also be able to use the methods we have described in this study to investigate whether changes in survival among cancer patients or other groups of patients may be attributable to changes in demographics, stage distribution, treatment mix, or technology.

## Conclusion

Five-year CRC survival rates improved between 1992 and 2000. Changes in demographic characteristics of patients diagnosed with CRC worked against their survival, while technological advances have made the largest contribution towards improvement in survival during the study period. Our findings will be useful in shaping policy discussions regarding CRC treatment and screening, and our novel methods may enable future researchers to disentangle the various complicated influences on survival.

## Competing interests

This study was sponsored by a grant from GE Healthcare, Waukesha, WI.

Kathleen Lang, Jonathan R. Korn, Lisa M. Lines, and Joseph Menzin received research funding from GE Healthcare.

David W. Lee is an employee of GE Healthcare.

Craig C. Earle is a consultant for Boston Health Economics.

## Authors' contributions

KL designed the research methods and collected and analyzed the data. JRK designed the research methods and collected and analyzed the data. DWL designed the research methods. LML designed the research methods and collected and analyzed the data. JM designed the research methods. All authors contributed to data interpretation, made substantive contributions to the manuscript, and had final approval of the article.

## Pre-publication history

The pre-publication history for this paper can be accessed here:

http://www.biomedcentral.com/1471-2407/9/227/prepub

## Supplementary Material

Additional file 1 Table 1. Click here for file

Additional file 2 Table 2. Click here for file

Additional file 3 Table 3. Click here for file
