# Supplementary material for: Factors associated with improved survival among older colorectal cancer patients in the US: a population-based analysis
Source: BMC Cancer. 2009 Jul 13;9:227. doi: 10.1186/1471-2407-9-227 (PMC2717120; doi:10.1186/1471-2407-9-227)
Supplement: Additional file 1 — Table 1. [file 1471-2407-9-227-S1.doc]

Table 1. Administrative codes used to identify treatments

| **Type of treatment** | **ICD-9-CM Procedure** | **ICD-9-CM Diagnosis** | **HCPCS/CPT** | **Revenue Center** | **SEER Variable** |
| --- | --- | --- | --- | --- | --- |
| Chemotherapy | 99.25 | V58.1, V66.2, V67.2 | 964xx, 965xx, Q0083-Q0085, 51720, J7150, J8510, J8520, J8521, J8530-J8999, J9000-J9999 |  | N/A |
| Radiotherapy | 92.21-92.29 | V58.0, V66.1, V67.1 | 77401-77499, 77750-77799 | 0330, 0333 | rad1 from 1 through 6 |
| Surgery | 45.3x-45.9x |  |  |  | nosrg1 = 0 |
