# Supplementary material for: Factors associated with improved survival among older colorectal cancer patients in the US: a population-based analysis
Source: BMC Cancer. 2009 Jul 13;9:227. doi: 10.1186/1471-2407-9-227 (PMC2717120; doi:10.1186/1471-2407-9-227)
Supplement: Additional file 2 — Table 2. [file 1471-2407-9-227-S2.doc]

Table 2. Baseline and demographic characteristics of patients with CRC

| **Variable** | **Colon Cancer Patients** | **Rectal Cancer Patients** | **Combined CRC Cohort** |
| --- | --- | --- | --- |
| N | 37,808 | 13,619 | 51,427 |
| Age |  |  |  |
| Mean | 77.5 | 76.5 | 77.2 |
| Standard deviation | 7.0 | 6.8 | 7.0 |
| Median | 77.0 | 76.0 | 77.0 |
| IQR | 72-82 | 71-81 | 72-82 |
| Female | 57.1% | 49.5% | 55.1% |
| Race |  |  |  |
| White | 86.3% | 87.0% | 86.5% |
| African American | 7.7% | 6.0% | 7.2% |
| Hispanic | 0.9% | 1.1% | 1.0% |
| Other | 5.1% | 5.9% | 5.3% |
| Geographic region |  |  |  |
| Northeast | 17.0% | 17.1% | 17.0% |
| Midwest | 31.4% | 31.7% | 31.5% |
| West | 43.5% | 43.9% | 43.6% |
| South | 8.2% | 7.3% | 7.9% |
| Location of residence |  |  |  |
| Metropolitan county | 83.2% | 82.5% | 83.0% |
| Non-metropolitan county | 16.8% | 17.5% | 17.0% |
| Year of CRC diagnosis |  |  |  |
| 1992 | 11.1% | 11.1% | 11.1% |
| 1993 | 10.4% | 10.8% | 10.5% |
| 1994 | 10.2% | 10.5% | 10.3% |
| 1995 | 10.1% | 9.6% | 9.9% |
| 1996 | 9.8% | 9.6% | 9.7% |
| 1997 | 10.1% | 9.5% | 9.9% |
| 1998 | 9.9% | 10.0% | 10.0% |
| 1999 | 9.6% | 9.7% | 9.6% |
| 2000^c^ | 18.9% | 19.2% | 19.0% |
| Stage at diagnosis |  |  |  |
| Stage I | 22.9% | 32.3% | 25.4% |
| Stage II | 35.0% | 25.3% | 32.4% |
| Stage III | 24.3% | 21.7% | 23.6% |
| Stage IV | 17.7% | 20.7% | 18.5% |
| Charlson score^a^ |  |  |  |
| Mean (±SD) | 2.0 | 1.8 | 1.9 |
| Standard deviation | 1.9 | 1.8 | 1.9 |
| Median | 2.0 | 1.0 | 1.0 |
| IQR | 0-3 | 0-3 | 0-3 |
| Selected Charlson comorbidities |  |  |  |
| Chronic pulmonary/respiratory disease | 33.1% | 33.1% | 33.1% |
| Congestive heart failure | 34.4% | 30.0% | 33.2% |
| Diabetes without complications | 25.8% | 23.9% | 25.3% |
| Cerebrovascular disease | 24.2% | 21.5% | 23.5% |
| Myocardial infarction | 15.6% | 14.1% | 15.2% |
| Peptic ulcer | 10.7% | 8.2% | 10.0% |
| Other major conditions^b^ | 0.0% | 0.0% | 0.0% |

Source: SEER-Medicare data, 1992-2005.

^a^Modified Charlson comorbidity index[16] excluding cancer-related comorbidities;

^b^Other major conditions include rheumatologic disease, mild liver disease, diabetes with complications, major liver disease peripheral vascular disease, dementia, renal disease, hemiplegia or paraplegia, and AIDS

^c^The higher proportion of patients diagnosed in 2000 is attributable to an expansion of the number of registries included in SEER.
