# Supplementary material for: Factors associated with improved survival among older colorectal cancer patients in the US: a population-based analysis
Source: BMC Cancer. 2009 Jul 13;9:227. doi: 10.1186/1471-2407-9-227 (PMC2717120; doi:10.1186/1471-2407-9-227)
Supplement: Additional file 3 — Table 3. [file 1471-2407-9-227-S3.doc]

Table 3. Logistic regressions of 5-year observed survival

|  | **Colon Cancer** | | | | **Rectal Cancer** | | |
| --- | --- | --- | --- | --- | --- | --- | --- |
| **Independent Variable** | **OR** | **95% CI** | ***P* value** | **OR** | | **95% CI** | ***P* value** |
| Age (vs. 85+) |  |  |  |  | |  |  |
| Age 65-74 | 3.987 | 3.693-4.304 | **<.001** | 5.082 | | 4.373-5.908 | **<.001** |
| Age 75-84 | 2.334 | 2.173-2.505 | **<.001** | 2.915 | | 2.518-3.374 | **<.001** |
| Female (vs. male) | 1.322 | 1.258-1.390 | **<.001** | 1.324 | | 1.217-1.440 | **<.001** |
| White (vs. non-white) | 1.063 | 0.984-1.147 | .120 | 1.060 | | 0.928-1.211 | .389 |
| Region (vs. Northeast) |  |  |  |  | |  |  |
| Midwest | 1.171 | 1.084-1.265 | **<.001** | 1.036 | | 0.908-1.181 | .599 |
| West | 1.141 | 1.064-1.224 | **<.001** | 1.059 | | 0.938-1.195 | .354 |
| South | 0.946 | 0.850-1.054 | .317 | 0.920 | | 0.758-1.115 | .395 |
| ≤ 25% of residents in ZIP code with < 12 years of education (vs. >25%) | 1.123 | 1.047-1.204 | **.001** | 1.182 | | 1.052-1.329 | **.005** |
| Median household income of ZIP code ≤ $50,000 (vs. >$50,000) | 0.924 | 0.872-0.979 | **.008** | 0.872 | | 0.790-0.964 | **.007** |
| Metropolitan county (vs. non-metropolitan) | 0.855 | 0.797-0.918 | **<.001** | 0.859 | | 0.763-0.966 | **.011** |
| Treatment* |  |  |  |  | |  |  |
| CT only, RT only, or CT and RT only | 10.758 | 6.784-17.060 | **<.001** | - | | - | **-** |
| Surgery only | 18.596 | 12.010-28.795 | **<.001** | 10.363 | | 7.703-13.941 | **<.001** |
| Surgery and CT | 30.482 | 19.592-47.426 | **<.001** | 15.570 | | 11.200-21.646 | **<.001** |
| Surgery, CT, and RT | - | - | **-** | 13.292 | | 9.756-18.110 | **<.001** |
| Surgery and RT | - | - | **-** | 10.310 | | 7.500-14.173 | **<.001** |
| Charlson score^a^ (vs. Charlson score of 0) |  |  |  |  | |  |  |
| Charlson score of 1 | 0.610 | 0.571-0.651 | **<.001** | 0.635 | | 0.562-0.718 | **<.001** |
| Charlson score of 2 or more | 0.301 | 0.277-0.327 | **<.001** | 0.304 | | 0.259-0.358 | **<.001** |
| Year (vs. 1992) |  |  |  |  | |  |  |
| 1993 | 1.010 | 0.909-1.121 | .856 | 1.020 | | 0.857-1.214 | .826 |
| 1994 | 1.021 | 0.920-1.134 | .691 | 1.041 | | 0.873-1.242 | .654 |
| 1995 | 0.984 | 0.885-1.093 | .762 | 1.089 | | 0.909-1.305 | .355 |
| 1996 | 1.171 | 1.053-1.303 | **.004** | 1.165 | | 0.972-1.397 | .099 |
| 1997 | 1.147 | 1.032-1.275 | **.011** | 1.210 | | 1.009-1.450 | **.039** |
| 1998 | 1.192 | 1.073-1.324 | **.001** | 1.228 | | 1.027-1.467 | **.024** |
| 1999 | 1.270 | 1.142-1.413 | **<.001** | 1.453 | | 1.211-1.742 | **<.001** |
| 2000 | 1.345 | 1.225-1.476 | **<.001** | 1.382 | | 1.180-1.617 | **<.001** |
| Stage (vs. Stage I) |  |  |  |  | |  |  |
| Stage II | 0.620 | 0.583-0.659 | **<.001** | 0.516 | | 0.466-0.573 | **<.001** |
| Stage III | 0.238 | 0.221-0.256 | **<.001** | 0.277 | | 0.246-0.311 | **<.001** |
| Stage IV | 0.015 | 0.013-0.017 | **<.001** | 0.022 | | 0.017-0.028 | **<.001** |

Source: SEER-Medicare data, 1992-2005.

^a^Reference group for CC patients is no treatment. Reference group for RC patients is no treatment OR CT only, RT only, or CT and RT only

^b^Modified Charlson comorbidity index[16]

Notes: Bold indicates statistical significance. Model compares odds of surviving 5 years, adjusted for age, sex, race, region, income, education (defined at the ZIP code level), urban/rural residence (defined by metropolitan statistical area), type of treatment, stage at diagnosis, and year of diagnosis.
